# Lost in Transition: HIV Prevalence and Correlates of Infection among Young People Living in Post-Emergency Phase Transit Camps in Gulu District, Northern Uganda

**DOI:** 10.1371/journal.pone.0089786

**Published:** 2014-02-28

**Authors:** Sheetal Patel, Martin T. Schechter, Nelson K. Sewankambo, Stella Atim, Noah Kiwanuka, Patricia M. Spittal

**Affiliations:** 1 School of Population and Public Health, University of British Columbia, Vancouver, British Columbia, Canada; 2 Centre for Health Evaluation and Outcome Sciences, Vancouver, British Columbia, Canada; 3 Makerere University College of Health Sciences, Kampala, Uganda; 4 Community-based Researcher, Gulu Town, Uganda; 5 School of Public Health, Makerere University College of Health Sciences, Kampala, Uganda; Banaras Hindu University, India

## Abstract

**Objective:**

Little is known about HIV infection and the related vulnerabilities of young people living in resource-scarce, post-emergency transit camps that are now home to thousands of Internally Displaced Persons (IDPs) following two decades of war in northern Uganda. The objective of this analysis was to assess the prevalence and correlates of HIV infection among young people living in post-conflict transition in Gulu District, northern Uganda.

**Methods:**

In 2010, a cross-sectional demographic and behavioural survey was conducted in two of Gulu District’s sub-counties with 384 purposively selected transit camp residents aged 15 to 29 years. Biological specimens were collected for rapid HIV testing in the field and confirmatory laboratory testing. Multivariable logistic regression identified independent determinants of HIV infection.

**Results:**

HIV prevalence was alarmingly high at 12.8% (95% CI: 9.6%, 16.5%). The strongest determinant of HIV infection among young people was a non-consensual sexual debut (adjusted odds ratio [AOR], 9.88; 95% CI: 1.70–18.06). Residing in Awach sub-county (AOR, 2.93; 95% CI: 1.28–6.68), experiencing STI symptoms in the previous 12 months (AOR, 2.36; 95% CI: 1.43–6.17), and practicing dry sex (AOR, 2.31; 95% CI: 1.04–5.13) were other key determinants of HIV infection.

**Conclusions:**

Study findings contribute to filling an important gap in epidemiological evidence and are useful for planning public health interventions in northern Uganda that effectively target young people in post-conflict transition and support them in the resettlement process. Findings serve to recommend reaching beyond traditional prevention programming in a way more effectively beneficial to young people in post-conflict settings by developing population-specific responses sensitive to local contexts and sufficient to address the underlying causes of the complex risk factors influencing the spread of HIV.

## Introduction

For over two decades prior to 2006, northern Uganda experienced a brutal conflict between the Government of Uganda and the rebel force, the Lord’s Resistance Army (LRA). More than 1.8 million people (which accounted for over 90% of the affected population) were forcibly displaced into Internally Displaced People (IDP) camps and were entirely dependent upon relief aid and services [1]–[5]. Nearly 70 percent of the displaced population were under 25 years of age [6] and an estimated 66,000 young people between the ages of 14 to 30 years had been abducted by the rebels and forced to serve as child soldiers, labourers and sex slaves [7]. The members of this traumatized generation are possibly at a heightened risk of contracting HIV.

In 2001, the United Nations General Assembly Special Session on HIV/AIDS passed the Declaration of Commitment on HIV/AIDS, stating that “populations destabilized by armed conflict, including refugees, internally displaced persons and in particular women and children, are at increased risk of exposure to HIV infection” [8]. The well-documented reasons for this include a breakdown in social structures; inadequate income; unmet basic needs; sexual violence; increased drug use, and; a lack of health and education infrastructures [9]–[13]. Spiegel (2004) also acknowledges that reduced mobility, isolated locations, and humanitarian aid might decrease displaced populations’ vulnerability to HIV [9]. The cases of South Sudan, Sierra Leone, Angola, and eastern DRC demonstrate that conflict can both enable HIV transmission and protect people from it [9], [14]–[18].

However, far less is known about the post-conflict period and its implications for HIV transmission, including the risk and risk behaviours of young people living in post-emergency phase transit camps. Shortly after the LRA and the Government of Uganda signed the Cessation of Hostilities Agreement in August 2006, northern Uganda’s IDPs were encouraged to move back to their ancestral homes or to smaller transit camps near their home villages. More than 90 per cent of the displaced population had returned home by 2011, while approximately 182,000 IDPs remain in transit camps [19]. The transit camp population should be analyzed independently since prolonged displacement and a lack of essential services and necessities combined with increased mobility within the post-conflict region, could exacerbate HIV transmission - as occurred in Mozambique and Afghanistan - undermining the redevelopment process [9], [20]–[23].

Humanitarian efforts during the LRA conflict were relief-focused and HIV/AIDS was not a high priority [24]–[27]. Consequently, HIV epidemiology for northern Uganda is severely limited. While NGOs have consistently reported that HIV may be devastating the region, neither antenatal studies [28]–[32] nor the Ugandan HIV/AIDS Sero-Behavioural Survey [33] – the only sources of HIV epidemiological data – identify the most vulnerable groups or capture the conflict’s legacy on HIV [25]–[27]. Post-conflict programme planners with limited funding for HIV/AIDS are therefore unable to assess who is most at risk for HIV infection, which is critical to the design, implementation and evaluation of interventions in resource scarce post-conflict settings [21].

This study aimed to help fill the gap in epidemiological evidence by providing an accurate understanding of the magnitude and determinants of HIV infection among young people undergoing post-conflict transition in Gulu District, northern Uganda. Our goal was to assist the Government of Uganda and NGOs in establishing and prioritizing effective post-conflict responses to HIV among young people. Moreover, analyzing the social, biological, and behavioural factors associated with infection will generate new insights into HIV transmission in post-conflict settings.

## Methods

### Ethics Statement

Ethics approval for this study was obtained from the Providence Healthcare Research Ethics Board, University of British Columbia, Canada; the Research and Ethics Committee, Child Health and Development Centre, College of Health Sciences, Makerere University, Uganda; the Uganda National Council of Science and Technology, and; the Republic of Uganda, Office of the President.

### Study Team

Fieldwork for this study occurred between May-December 2010 and commenced with the hiring and training of a 15-person research team, consisting of: four research assistants (two male, two female); one nurse; one HIV/AIDS counsellor; two out-reach trauma counsellors (one male and one female); one laboratory technician; four community mobilizers (two in each research site), and two drivers. All team members were Acholi and bilingual in the local language of Luo and English, had worked extensively in the region before, and underwent a month-long intensive training and preparation session.

### Study Design and Sampling

A cross-sectional study design was employed to determine the prevalence and correlates of HIV infection among 15- to 29-year-olds residing in Gulu District transit camps. Two sub-counties (Awach and Ongako) were randomly selected from all Gulu District sub-counties with transit camps (i.e., 18/23 sub-counties). Twelve transit camps from Awach and Ongako (ranging from remote to accessible with varying degrees of resource availability) were purposively selected, with the guidance of key informants. A sample was drawn by combining proportional and non-proportional quota sampling methods, as random and systematic methods were not feasible in the transit camps due to limited data on the population and the camps’ unsystematic layouts. We collaborated with hired community members who mobilized prospective participants according to the study’s eligibility criteria: 1.) aged 15 to 29 years; 2.) resident in an Awach or Ongako transit camp, and; 3.) provision of consent (and parental assent when applicable) for participation. Of the 385 individuals mobilized for the study, 384 agreed to participate.

#### Sample size

The sample size required to estimate a 10% prevalence rate of HIV among young people - with a precision of ±3% (with 95% confidence) – was calculated to be 384. The sample was proportional to the population size of each sub-county: 216 participants for Ongako (1.50% of 14,360 people) and 168 for Awach (1.50% of 11,160). Additionally, we employed a non-proportional quota of 50% male and 50% female.

### Data Collection

#### Informed consent process

Our research team first asked heads of household for permission to speak with and enroll age-eligible household members in our study. If permission was granted, the study and consent forms were then thoroughly explained to the participant in the local language of Luo. Written or thumbprint informed consent was obtained. Minors under 18 also required a parent/guardian’s consent for participation in our study (if the parent or guardian consented but the minor did not provide assent, that minor was not enrolled in our study), unless they were emancipated. The Ugandan National Council for Science and Technology defines anyone under the age of 18 who is married, has children or is currently pregnant as an emancipated minor who may independently provide informed consent to participate in research [34].

#### Questionnaires and HIV testing

A demographic and behavioural questionnaire with blood specimen collection was used to determine HIV prevalence and collect information on the socio-demographic characteristics, conflict-related experiences and sexual behaviours of young people. Each respondent was interviewed by a same-sex Acholi research assistant who was blind to participants’ HIV status and fluent in Luo and English. Following the interview, a trained nurse collected blood samples. The INSTI HIV-1/HIV-2 Antibody Rapid HIV Test (bioLytical Laboratories) was administered and an additional sample of blood was drawn through venipuncture from those who tested positive for confirmatory laboratory testing. Serum samples were tested for HIV-specific antibodies using an enzyme-linked immunosorbent assay (ELISA), the Abbott Murex HIV-1/2 ELISA (Murex Biotech Limited, United Kingdom). Reactive specimens were confirmed using a second ELISA test, the Vironostika HIV Uni-Form II MicroELISA (bioMerieux, Switzerland). Samples were considered positive when both ELISA tests were positive. Any sample yielding discordant ELISA test results was subjected to Western Blot analysis (Calypte Biomedical) for definitive characterization.

Interviews and testing were conducted anonymously at spaces chosen by respondents in their homes. Adhering to Ugandan HIV counselling and testing policy guidelines, all participants received pre- and post-test counselling and had the option of receiving their test results. As the interviews and/or tests could precipitate distress, immediate psychosocial support and referrals for follow-up HIV care were made available. In addition, participants received 4000 UG Shillings each (∼$2USD) as compensation for their time.

### Statistical Analysis

All analyses were conducted using SPSS Version 19.0. Point estimates of HIV prevalence and corresponding 95% confidence intervals were calculated separately for: all participants; female and male participants; and participants who had and had not been abducted by the LRA. All variables were described using summary statistics and a bivariate analysis was conducted to examine socio-demographic and behavioural differences between HIV-positive and HIV-negative participants. Categorical variables were compared using Pearson’s Chi-Square; when 25% or more of the expected cell frequencies in a contingency table were less than five, Fisher’s Exact Test was used. For continuous variables, an Independent Samples T-test compared the means of normally distributed variables between HIV-positive and HIV-negative participants, while the Wilcoxon Rank-Sum Test compared medians of non-normally distributed variables. Logistic regression was used to determine independent risk factors for HIV infection among sexually active participants. Variables were included for unadjusted regression analysis based on significance at the *p*≤0.05 level in bivariate analysis. The multivariable regression model adjusted for all variables that were statistically associated with HIV infection at a significance level of *p*≤0.05 in unadjusted regression analyses as well as ‘sex’. Since ‘ever married’ and ‘school status’ were correlated, only school status was entered into the multivariable model as it was hypothesized *a priori* to have a protective association with HIV infection. Similarly, ‘ever having been raped’ and ‘non-consensual sexual debut’ were correlated, so only the latter variable was entered into the regression model. Odds ratios (OR) with corresponding 95% confidence intervals (CI) were calculated and all reported *p*-values are two-sided.

## Results

### Socio-demographic Characteristics and Background Information

Participants’ median age was 20 years and 50.0% of respondents were female (Table 1). Over 80% were Roman Catholic and most were Acholi. All participants lived in transit camps (median distance of 7 km from their home village). Over 40% had lived there for 1 to 2 years and nearly 25% anticipated staying another 1 to 2 years. Five percent of respondents were living in child-headed households and 60.4% had ever married. The median age at first marriage was 17 years and 43.1% of the marriages were polygynous (more than one wife). A quarter of the participants were currently attending school while more than 70% had dropped out at some point, primarily due to poverty (i.e., lack of money for school fees). Seventy-one percent of participants were subsistence farmers, 9.4% did petty trades/casual labour, and 3.1% brewed alcohol as their main means of livelihood. Sixty percent of respondents earned less than 25,000 Ugandan shillings (∼$12USD) monthly. Forty percent of participants currently lacked food and/or water, while more than 50% had gone hungry in the previous year.

**Table 1:** Socio-demographic characteristics of study participants (N = 384).

Characteristic	Sub-characteristic	No. (%) of participants
Age	15–19 years	172 (44.8)
	20–24 years	98 (25.5)
	25–29 years	114 (29.7)
	Median (range)	20 (15–19)
Sex	Female	192 (50.0)
	Male	192 (50.0)
Sub-county	Awach	168 (43.8)
	Ongako	216 (56.2)
Religion	Roman Catholic	311 (81.0)
	Protestant Church of Uganda	47 (12.2)
	Muslim	14 (3.7)
	Pentecostal Christian	12 (3.1)
Tribe	Acholi	376 (97.9)
	Other	8 (2.1)
Distance to village of origin, km, median (range)	-----	7 (0–100)
Duration of stay in transit camp	1–6 months	20 (5.2)
	7–12 months	65 (16.9)
	13 months-2 years	166 (43.2)
	3–5 years	97 (25.3)
	More than 5 years	36 (9.4)
Duration of time left living in transit camp	1–11 months	185 (48.2)
	1–2 years	92 (23.9)
	3–8 years	30 (7.8)
	Forever	13 (3.4)
	Don’t know	64 (16.7)
Living arrangements	Child-headed household	19 (4.9)
	Other	365 (95.1)
Marital status	Currently married	150 (39.0)
	Other	82 (21.4)
	Never married	152 (39.6)
Age at first marriage, yr, median (range)	-----	17 (9–25)
Polygynous marriage	-----	100 (43.1) n = 232
School status	Ever in school	374 (97.4)
	Currently in school	96 (25.0)
	Dropped out	278 (72.4)
Reason for school drop-out	No money for school fees	174 (62.5)
	Got married	21 (7.6)
	Left school during war	30 (10.8)
	Pregnancy (females only)	30 (10.8)
	Other	23 (8.3)
Main means of livelihood	Subsistence farmer	271 (70.6)
	Petty trade/Casual labour	36 (9.4)
	Brew alcohol	12 (3.1)
	Other	65 (16.9)
Monthly income <25,000 UGS^1^	-----	232 (60.4)
Currently experience lack of food and/or water	-----	153 (39.8)
Not enough food to eat past 12 months	-----	207 (53.9)
Duration of stay in IDP camps	Never	12 (3.1)
	3–5 years	28 (7.3)
	5–10 years	85 (22.1)
	More than 10 years	221 (57.6)
	Since I was born	38 (9.9)
Abducted by the Lord’s Resistance Army	-----	107 (27.9)
	Female abductees	42 (39.3)
	Male abductees	65 (60.7)
Age at first abduction, yr, median (range)	-----	13 (6–21)
Ever night commute^2^	-----	278 (72.4)

1 Approximately $12USD.

2 Leaving your family hut at night to sleep elsewhere due to security and privacy concerns.

Three percent of respondents had never lived in an IDP camp, but 259 (67.4%) had lived in IDP camps for more than 10 years; 38 of whom were born there. Nearly 30% of participants had been abducted by the LRA (median age at first abduction was 13 years). Night-commuting during the war – movement at night to sleep elsewhere than your family hut for security and privacy – was common (72.4%).

### Risk Behaviour and Risk Perception

Seventy-eight percent of participants reported ever having had sex (Table 2). The median age at first sexual intercourse was 16 years, although 69 participants (22.9%) indicated having had sex before the age of 15 years. Over 17% of participants’ first sexual experience was not consensual and less than 40% used a condom on that occasion. Seventeen percent of participants’ first sexual partners were 10 or more years older than them. Sixty percent of respondents’ first sexual partners were students and 9.0% were military/rebel soldiers.

**Table 2:** Risk behaviour and risk perception of study participants (N = 384).

Characteristic	Sub-characteristic	No. (%) of participants
Sexually active (ever)	-----	301 (78.4)
Age at first sex, yr, median (range)	-----	16 (6–25)
Age at first sex less than 15 yrs. old	-----	69 (22.9)
Non-consensual sexual debut^a^	-----	52 (17.3)
Condom use at first sex^a^	-----	113 (37.5)
First sex partner 10 or more years older^a^	-----	51 (16.9)
Main occupation of first sex partner^a^	Student	179 (59.5)
	Subsistence farmer	60 (19.9)
	Military/Rebel soldier	27 (9.0)
	Other	35 (11.6)
Currently have sex partner besides your spouse^b^	-----	17 (11.3)
Sexually active past 12 months^a^	-----	234 (77.7)
Symptoms of STI past 12 months^c^	-----	63 (26.9)
Sexually active past 6 months^a^	-----	207 (68.8)
Had sex partner(s) from outside your community past 6 months	-----	26 (12.6) n = 207
Ever consumed alcohol	-----	58 (15.1)
Age began drinking alcohol, yr, median (range)	-----	20 (13-27)
Ever consumed alcohol before sex^a^	-----	29 (9.6)
Consumed alcohol before last sex	-----	15 (51.7) n = 29
Ever practice dry sex^1^^,^ a	-----	152 (50.5)
Practice dry sex last sexual encounter^a^	-----	152 (50.5)
Ever survival sex work^2^^,^ a	-----	11 (3.7)
Age at first survival sex experience, yr, median (range)	-----	17.5 (14–28)
Age of partner at first survival sex experience, yr, median (range)	-----	48 (17–53)
Ever experience physical/sexual/verbal abuse from sexual partner^a^	-----	150 (49.8)
Ever been raped^a^	-----	59 (19.6)
Age at rape, yr, median (range)	-----	14 (6–24)
Perpetrator 10 or more years older^d^	-----	35 (59.3)
Main occupation of prepetrator^d^	Military/Rebel soldier	19 (32.2)
	Subsistence agriculturalist	15 (25.4)
	Student	12 (20.3)
	Other	13 (22.0)
Easy to get condoms in area	-----	242 (63.0)
Never had condom demonstration	-----	155 (40.4)
Ever used condom	-----	208 (54.2)
Condom used last sex^a^	-----	76 (25.2)
Consistency of condom use^e^	Always	56 (26.9)
	Sometimes or never	152 (73.1)
Ever HIV test	-----	314 (81.8)
No. HIV tests in lifetime, median (range)	-----	14 (0-30)
Know partner’s HIV status^a^	-----	162 (53.8)
Can protect self from HIV/STIs	-----	320 (83.3)
Able to say no to sex	-----	334 (87.0)
Very likely been exposed to HIV	-----	49 (12.8)

1 Sexual intercourse without foreplay or lubrication so that the vagina is dry upon penetration.

2 Exchaning sex for food, shelter, money, gifts.

a Among those reporting ever having had sex (*n* = 301).

b Among those who are currently married (*n* = 150).

c Among those reporting sexual activity in past 12 months (*n* = 234).

d Among those reporting ever having been raped (*n* = 59).

e Among those reporting ever having used a condom (*n* = 208).

Nearly 80% of sexually active respondents reported having had sex in the previous year and 26.9% of those had experienced STI symptoms during that time. Ten percent of sexually active participants had consumed alcohol before sex and of those, 51.7% had done so before their last sexual experience. The practice of dry intercourse – without foreplay or lubrication and with the aid of drying agents – was common and reported by 50.5% of participants. It served to increase men’s sexual pleasure (79.3%) and allow women to express fidelity (92.6%). Eleven (3.7%) of the sexually active participants – all female – reported survival sex work for food, shelter, money or gifts. The median age at first survival sex experience was 17.5 years and the median age of partner was 48 years.

Half of all participants had been physically, sexually or verbally abused by a sexual partner, while 19.6% had been raped. The median age at rape was 14 years and almost 60% of the perpetrators were 10 or more years older than their victims. Over 30% of the perpetrators were military or rebel soldiers, while 25.4% were subsistence agriculturalists.

Sixty-three percent of respondents had easy access to free condoms, yet 40.4% (155) had never seen condom use demonstrated. Over 50% of participants had used a condom previously, but only 25.2% had used a condom during their last sexual encounter. Only 26.9% of condom users reported consistent use (i.e., always vs. sometimes or never). Over 80% of participants indicated having had an HIV test at least once and the median number of times tested for HIV in lifetime was 14. Over half of sexually active participants (53.8%) were aware of their partner’s HIV status. Almost 85% of respondents thought they could protect themselves from HIV/STIs; 66.7% of these believed that testing blood with their partner before sex most effectively prevented infection. Nearly 13% of respondents considered it very likely that they had been exposed to HIV.

### Prevalence of HIV Infection

As seen in table 3, 49 of 384 participants tested positive for the HIV antibody, yielding an overall prevalence rate of 12.8% (95% CI: 9.6%, 16.5%). HIV prevalence was 15.6% (95% CI: 10.8%, 21.6%) among females, 9.9% (95% CI: 6.1%, 15.0%) among males, 12.1% among formerly abducted participants, and 13% (95% CI: 9.3%, 17.5%) among non-abducted participants.

**Table 3:** Prevalence of HIV infection among study participants.

	Prevalence Estimate (%)
	[95% CI]
Category	*(# Infected/Total N)*
All participants	12.8
	[9.6–16.5]
	*(49/384)*
Females	15.6
	[10.8–21.6]
	*(30/192)*
Males	9.9
	[6.1–15.0]
	*(19/192)*
Formerly Abducted	12.1
	[6.6–19.9]
	*(13/107)*
Non-abductees	13.0
	[9.3–17.5]
	*(36/277)*

### Comparisons Between HIV-Positive and HIV-Negative Participants

As seen in table 4, our bivariate analysis of socio-demographic characteristics demonstrated positive associations between HIV infection and: older age (26 vs. 20 years, *p*<0.001); residing in Awach sub-county (67.3% vs. 40.3%, *p*<0.001); having been married (91.8% vs. 55.8%, *p*<0.001); currently lacking food and/or water (57.1% vs. 37.3%, *p* = 0.008), and; living in an IDP camp for more than 10 years (79.6% vs. 65.7%, *p* = 0.052). HIV-positive participants were significantly *less likely* than HIV-negative participants to currently be in school (12.2% vs. 26.9%, *p* = 0.027) and have night-commuted during the war (59.2% vs. 74.3%, *p* = 0.027). While HIV infection was marginally associated with being female (*p* = 0.092), it was not associated with former abduction (*p* = 0.824).

**Table 4:** Comparison of socio-demographic and behavioural characteristics of HIV-positive (n = 49) and HIV-negative (n = 335) participants.

Variable	No. (%) of HIV-positive	No. (%) of HIV-negative	*p value*
*Socio-Demographics & Background Information*			
Age, yr, median (range)	26 (15–29)	20 (15–29)	<0.001
Female sex	30 (61.2)	162 (48.4)	0.092
Awach sub-county	33 (67.3)	135 (40.3)	<0.001
Ever married	45 (91.8)	187 (55.8)	<0.001
Currently in school	6 (12.2)	90 (26.9)	0.027
Current lack of food and/or water	28 (57.1)	125 (37.3)	0.008
Had enough food to eat past 12 months	18 (36.7)	159 (47.5)	0.159
Duration of stay in IDP camps >10 years	39 (79.6)	220 (65.7)	0.052
Formerly abducted	13 (26.5)	94 (28.1)	0.824
Ever night commute^1^	29 (59.2)	249 (74.3)	0.027
***Behavioural Characteristics***			
Age of sexual debut, yr, median (range)	16 (8–23)	16 (6–25)	0.451
Non-consensual sexual debut^a^	18 (37.5)	34 (13.4)	<0.001
First sex partner 10 or more years older^a^	16 (33.3)	35 (13.8)	<0.001
STI symptoms past 12 months^b^	20 (57.1)	43 (21.6)	0.002
Ever consumed alcohol	7 (14.3)	51 (15.2)	0.864
Ever consumed alcohol before sex^a^	4 (8.3)	25 (9.9)	0.987
Consumed alcohol before last sex^a^	4 (8.3)	11 (4.3)	0.272
Ever practice dry sex^2^^,^ a	31 (64.6)	121 (35.8)	<0.001
Ever survival sex work^3^^,^ a	3 (6.3)	8 (3.2)	0.154
Ever experience physical/sexual/verbal abuse^a^	27 (56.2)	123 (36.6)	0.014
Ever been raped^a^	18 (37.5)	41 (16.2)	<0.001
Perpetrator 10 or more years older^c^	13 (72.2)	22 (53.7)	0.115
Ever used a condom	28 (57.1)	180 (53.7)	0.654
Condom used last sex^a^	9 (18.8)	67 (20.0)	0.789
Consistent condom use^d^	8 (28.6)	48 (26.7)	0.605
No. HIV tests in lifetime, median (range)	5 (0–30)	15 (0–30)	0.001
Know partner’s HIV status^a^	14 (28.5)	148 (44.2)	0.039
Can protect self from HIV/STIs	26 (53.1)	294 (87.8)	<0.001
Able to say no to sex	39 (79.6)	295 (88.1)	0.100

1 Leaving your family hut at night to sleep elsewhere due to security and privacy concerns.

2 Sexual intercourse without foreplay or lubrication so that the vagina is dry upon penetration.

3 Exchanging sex for food, shelter, money, gifts.

a Among those reporting ever having had sex, HIV-positive (*n* = 48) HIV-negative (*n* = 253).

b Among those reporting sexual activity in past 12 months, HIV-positive (*n* = 35) HIV-negative (*n* = 199).

c Among those reporting ever having been raped, HIV-positive (*n* = 18) HIV-negative (*n* = 41).

d Among those reporting ever having used a condom, HIV-positive (*n* = 28) HIV-negative (*n* = 180).

Behavioural characteristics positively associated with HIV infection included: a non-consensual sexual debut (37.5% vs. 13.4%, *p*<0.001); first sex partner being 10 or more years older (33.3% vs. 13.8%, *p*<0.001); having STI symptoms in the past year (57.1% vs. 21.6%, *p* = 0.002); having practiced dry sex (64.6% vs. 35.8%, *p*<0.001); having been physically, sexually or verbally abused by a sexual partner (56.2% vs. 36.6%, *p* = 0.014), and; having been raped (37.5% vs. 16.2%, *p*<0.001). HIV-positive participants were significantly *less likely* than HIV-negative participants to think that they could protect themselves from HIV/STIs (53.1% vs. 88.8%, *p*<0.001) and to know their partner’s HIV status (28.5% vs. 44.2%, *p* = 0.039). Additionally, HIV-positivity was negatively associated with a greater median number of HIV tests taken in lifetime (5 vs. 15, *p* = 0.001)

### Factors Independently Associated With Prevalent HIV

In the multivariable regression model for sexually active participants (Table 5), HIV-positivity was independently associated with older age (AOR, 1.17; 95% CI: 1.05–1.32); place of residence (AOR, 2.93; 95% CI: 1.28–6.68 for Awach vs. Ongako sub-county); a non-consensual sexual debut (AOR, 9.88; 95% CI: 1.70–18.06); the practice of dry sex (AOR, 2.31; 95% CI: 1.04–5.13); experiencing STI symptoms in the past year (AOR, 2.36; 95% CI: 1.43–6.17); believing one can protect themselves from HIV/STIs (AOR, 0.29; 95% CI: 0.12–0.69), and; a greater number of HIV tests taken in lifetime (AOR, 0.86; 95% CI: 0.81–0.91).

**Table 5:** Determinants of HIV infection by logistic regression for sexually active participants (n = 301).

Variable	Unadjusted odds ratio [95% CI]	Adjusted odds ratio [95% CI]
Age	1.23 [1.14–1.33]	1.17 [1.05–1.32]
Female sex	1.69 [0.91–3.11]^a^	1.54 [0.54–4.43]
Awach sub-county	3.06 [1.62–5.77]	2.93 [1.28–6.68]
Currently in school	0.05 [0.01–0.39]	0.38 [0.04–3.92]
Currently experience lack of food and/or water	2.24 [1.22–4.11]	1.46 [0.62–3.42]
Duration of stay in IDP camps >10 years (ref = ≤10 years)	2.04 [0.98–4.23]	-----
Ever night commute^1^	0.50 [0.27–0.93]	0.69 [0.31–1.52]
Non-consensual sexual debut	5.14 [2.60–10.15]	9.88 [1.70–18.06]
First sex partner 10 or more years older (ref = same age)	3.05 [0.79–11.76]	-----
STI symptoms past 12 months	4.68 [2.44–9.00]	2.36 [1.43–6.17]
Ever practice dry sex^2^	3.05 [1.64–5.67]	2.31 [1.04–5.13]
Ever experience physical/sexual/verbal abuse	2.12 [1.16–3.87]	1.09 [0.48–2.46]
No. HIV tests in lifetime	0.94 [0.90–0.98]	0.86 [0.81–0.91]
Know partner’s HIV status	0.29 [0.15–0.54]	0.50 [0.22–1.12]
Can protect self from HIV/STIs	0.16 [0.08–0.30]	0.29 [0.12–0.69]

1 Leaving your family hut at night to sleep elsewhere due to security and privacy concerns.

2 Sexual intercourse without foreplay or lubrication so that the vagina is dry upon penetration.

a Forced entry.

## Discussion

It is unclear whether northern Uganda’s prolonged conflict has had a protective or enabling effect on HIV transmission. Understanding local epidemic patterns is therefore important for developing effective responses to HIV as former IDPs transition through the post-conflict process. Based on the HIV prevalence of 12.8% identified among young people in this study, Gulu District is a high prevalence District in the Ugandan context. Although there are no historic population-based statistics for 15- to 29-year-olds in Gulu District to reference, the Ugandan Sero-Behavioural Survey, last conducted in 2004/05, provides regional estimates of HIV infection among 15- to 49-year-olds. Its estimate for the whole North Central region is 8.2% [33], which suggests that Gulu District has a high prevalence within Uganda. In addition, estimates based on antenatal clinic (ANC) data collected in 2005 demonstrate HIV prevalence among pregnant women to be higher in Gulu District (10.3%) than the surrounding North Central Districts (9.1% in Kitgum, 4.3% in Pader), also indicating a high HIV prevalence in Gulu District [32]. Moreover, the recent Uganda Ministry of Health’s 2011 AIDS Indicator Survey estimated the national HIV prevalence rate to be 7.3% - almost 45% less than what we observed in Gulu District [35].

Our findings indicate that the risk for HIV infection may be greater in some parts of the District than others. We identified that participants residing in Awach sub-county were three times more likely to be HIV-positive than participants from Ongako sub-county. This differential in risk may be explained by the increased mobility of Awach residents due to the sub-county’s relatively remote location. Movement to and from neighbouring communities, to attend auctions and access mobile markets for household necessities, is common and may be a risk factor. Sixty-two percent of participants from Awach reported travel to an auction or mobile market in the past year, as compared to 35% of participants from Ongako. Further research examining the individual and community-level factors responsible for HIV prevalence differentials between sub-counties in Gulu District is required, so that preventative interventions can be directed to those areas most in need.

Other studies from Uganda corroborate our findings that older age, a non-consensual sexual debut and having experienced STI symptoms in the past year are significant risk factors for HIV infection [33], [36], [37]. However, our study is the first to have identified the practice of dry sex as a significant risk factor in Uganda. Participants who had practiced dry sex were more than twice as likely to be HIV-positive compared to those participants who had never practiced dry sex. All 152 (50.4%) participants who indicated ever having practiced dry sex also reported practicing dry sex at their last sexual encounter, illustrating the common frequency of this sexual custom. In the past two decades it has become increasingly apparent that dry sex is of critical importance to HIV transmission, particularly in areas heavily affected by HIV [38]–[40]. Dry sex reduces vaginal secretions containing lactobacilli, a natural defense against infection; lacerations in the vaginal wall are more likely to occur, increasing susceptibility to infection [40]. Further, recent research in Kenya, South Africa and Mozambique notes that people who practice dry sex may not use lubricated condoms or microbicides [38], [39], [41]–[46]. Likewise, 91.2% of the men in our study who had practiced dry sex had never used a condom before. Our findings signal the potential shortcomings of traditional HIV prevention programming in areas like Gulu District where dry sex is the norm, and highlight the importance of conducting further research (qualitative in nature) to identify which HIV prevention methods would be acceptable in these areas and investigating perspectives on actual product use. Concurrently, educating communities about the association between the practice of dry sex and an elevated risk of HIV infection must be a critical component of HIV prevention programming in northern Uganda.

Our study’s finding of a positive association between a non-consensual sexual debut and HIV infection also has important implications for the design and delivery of HIV prevention programming. A non-consensual first sexual experience was the strongest determinant of HIV infection among young people in this study and was reported by over 25% of young women and nearly 8% of young men. Despite growing recognition of the issue and its links to HIV infection, reproductive health and HIV prevention programmes for young people rarely address the reality of coercive sex and other sexual violence [47]. Traditional HIV prevention paradigms (i.e., the ABC strategy) exclusively emphasize individual behaviours such as abstinence, partner reduction, and condom promotion. As such, they fail to acknowledge social determinants of HIV (such as poverty, political instability, war, and gender inequality) that severely limit personal agency by diminishing young people’s capacity to choose whether or not to engage in sex and/or practice safe sex [24], [25]. Our finding calls attention to the importance of addressing the issue of sexual coercion as an integral component of HIV prevention programmes in post-conflict settings. However, a more holistic approach is needed that reaches beyond traditional prevention programming and: addresses any harmful gender norms that impact young people’s sexual behaviour and perpetuate male dominance and violence against women; helps young people to improve their communication and negotiation skills; strengthens legal and advocacy resources, and; trains health care professionals to improve clinical services for young people who have been sexually coerced or victim to sexual violence.

We also identified participants who reported experiencing STI symptoms in the past year as having a higher risk of HIV infection, as has been found in other African studies [37], [48], [49]. However, it is important to note that self-reported STI symptoms do not necessarily capture asymptomatic infections and may be subject to recall error. Nonetheless, given the synergistic role of STIs in HIV transmission, STI diagnosis and treatment must remain a priority in post-conflict HIV prevention programmes. To that end, health care workers should be trained in STI case detection and management using the syndromic approach, which can reduce HIV transmission considerably [11].

Our multivariable regression analysis also identified two protective associations with HIV infection. First, participants who believed that they could protect themselves from HIV/STIs were 71% less likely to be HIV-positive. A large number of these participants (89.3%) were in school, which supports the abundance of research demonstrating that schools can reduce HIV infection during conflict by teaching children about the disease, giving them access to HIV prevention interventions, keeping them safe during the day, and equipping them with life skills to protect themselves [50]–[52]. Although it is unclear whether the negative association between HIV-positivity and believing one can protect themselves from infection is the result of school-based HIV information and prevention programmes, our findings suggest that information campaigns and other HIV prevention efforts for general and school-going populations should be closely coordinated so that young people who are in and out of school have access to the same information and means to protect themselves. Second, we found that the probability of HIV infection decreases by 14% for every one-unit increase in the number of previous HIV tests taken. It is difficult to determine whether this is because of effective counselling and testing services or because individuals who learned they were HIV-positive stopped testing, while HIV-negative individuals continued to test. Further research is needed to determine the precise reason for this association and to explore the role that HIV testing may have to play in preventing infection in this population.

Our study had several limitations. The first limitation was the use of non-random sampling methods, which may have compromised the representativeness of our sample and thus the generalizability of our findings. Given that the majority of Gulu District’s population was encamped during the war, we believe that study participants are characteristically similar to other conflict-affected young people living in the District and our study results can therefore be generalized to this larger population. Second, our cross-sectional survey data was unable to identify cause-and-effect relationships. Third, the study’s identified HIV-positive-risk factor associations do not reveal relative temporal sequences. Finally, the self-reported data may be limited by social desirability bias leading to an underestimation of certain HIV risk behaviours.

## Conclusions

This study details the high magnitude of HIV infection among young people living in post-emergency phase transit camps in northern Uganda and characterizes the context-specific factors that contribute to the spread of infection. Study findings contribute to filling an important gap in epidemiological evidence and are useful for planning public health interventions that effectively target young people in post-conflict transition and support them in the resettlement process. The high HIV prevalence of 12.8% identified among young people in this study and the established key determinants of infection (a non-consensual sexual debut, the practice of dry sex, residing in Awach sub-county, and experiencing STI symptoms in the past year), urges for post-conflict HIV prevention and care interventions that are comprehensive in terms of coverage of vulnerable populations yet tailored to local circumstances in order to meet the distinct challenges that young people in post-conflict transition face. Developing such interventions will also benefit post-conflict societies generally. As young people are a significant proportion of these societies, they are vital to the recovery and reconstruction process. Confronting the realities of HIV can present a window of opportunity for strengthening the larger health development process and facilitating recovery. Indifference to the needs of young people ‘lost in transition’, however, does not bode well for the future of post-conflict societies.
